# *Rickettsia africae*, Western Africa

**DOI:** 10.3201/eid1603.090346

**Published:** 2010-03

**Authors:** Oleg Mediannikov, Jean-François Trape, Georges Diatta, Philippe Parola, Pierre-Edouard Fournier, Didier Raoult

**Affiliations:** Unité de Recherche sur les Maladies Infectieuses et Tropicales Emergentes, Marseille, France (O. Mediannikov, P. Parola, P.-E. Fournier, D. Raoult); Unité de Recherche sur les Maladies Infectieuses et Tropicales Emergentes, Dakar, Senegal (O. Mediannikov, J.F. Trape, G. Diatta)

**Keywords:** Rickettsia africae, Senegal, Amblyomma variegatum, spotted fever, African tick-bite fever, tick, rickettsia, letter

**To the Editor:** *Rickettsia africae*, the causative agent of African tick-bite fever, is transmitted by *Amblyomma hebraeum* and *A. variegatum* ticks (*1*,*2*). These ticks are common in western, central, and southern Africa. Adults rarely feed on humans, although nymphs attach more frequently and larvae are sometimes serious pests (abundant and aggressive) (*3*).

African tick-bite fever is a neglected disease that has been mainly detected in tourists who were bitten by a tick while traveling in disease-endemic areas (*2*). A recent worldwide report showed rickettsial infection incidence to be 5.6% in a group of travelers in whom acute febrile infection developed after they returned from sub-Saharan Africa. African tick-bite fever is the second most frequently identified cause for systemic febrile illness among travelers, following malaria (*4*). Seroprevalence for spotted fever group rickettsiae is high in the Sahel regions of Africa (*5*), although there may be different emergent and classic rickettsioses in Africa.

*R. africae* has been detected by PCR in many African countries, including Niger, Mali, Burundi, and Sudan (*6*), and in most countries of equatorial and southern Africa (Figure). Most strains and cases have been found in South Africa (*2*). *R. africae* and African tick-bite fever have not previously been reported in Senegal, and few positive human serum samples have been documented in western Africa. *A. variegatum*, the main vector of *R. africae*, was introduced by cattle into Guadeloupe, West Indies, from Senegal in the early 1800s. Spotted fever caused by *R. africae* has become endemic there in the past 30 years (*7*). In addition to *R. africae*, *A. variegatum* ticks may transmit other human and animal pathogens, including Crimean-Congo hemorrhagic fever virus, Dugbe virus, Thogoto virus, Bhanja virus, *Ehrlichia ruminantium, Theileria* spp., *Anaplasma* spp., and *Dermatophilus congolensis* (*3*,*6*).

**Figure:**
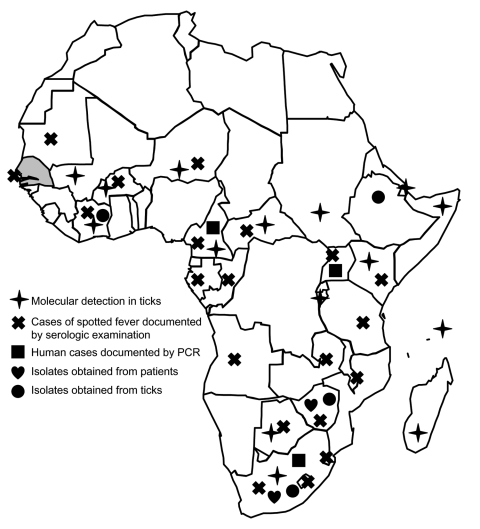
Distribution of *Rickettsia africae* in the African continent and serologic evidence of spotted fevers in humans. Gray shading indicates location of Senegal.

From November through December 2008, ticks were collected from domestic animals (cattle, goats, sheep, dogs, horses, donkeys) in the Sine-Saloum region of Senegal (villages Dielmo, Ndiop, Medina, and Passi). Among the collected ticks, 8 fully engorged nymphs were kept alive in flasks at 90%–95% relative humidity. Other ticks were stored in 70% ethanol. Flagging at ground level was used to collect ticks from pastures. Species were identified according to standard taxonomic keys for adult ticks. Nymphs were allowed to molt before identification and subsequent bacterial culture. Rickettsial DNA in other ticks was detected by semiquantitative PCR with *Rickettsia*-specific primers (*8*). All positive samples were subjected to PCR by using primers designed for the *gltA* and *ompA* genes (*6*). Three rickettsial spacers were chosen for typing: *dksA-xerC*, *rpmE-tRNAmet*, and *mppA-purC* (*9*).

Tick larvae were the only stage collected by flagging at ground level. Flagging for 30 minutes collected 495 larvae near the village of Passi and 325 in Dielmo. The larvae were aggressive, and several attached onto the collector’s ankles despite preventive measures. All larvae were morphologically identified as *Amblyomma* spp. Amplification and sequencing of the portion of mitochondrial cytochrome oxidase I gene of 3 adult *A. variegatum* ticks, 2 individual larvae, and 1 pool of 10 larvae detected a 659-bp sequence 100% identical among all larvae and adults and corresponding to cytochrome oxidase I of other ticks. The sequence is deposited in GenBank, accession no. GU062743.

Adult ticks (n = 492) were collected from domestic animals; 85 (17.3%) were *A. variegatum,* and 74 (87.1%) were positive for rickettsial genes according to real-time PCR. No associations between animal host, place of collection, and presence of *R. africae* were found (data not shown). During the subsequent amplification and sequencing of the 632-bp fragment of the *ompA* gene, all amplicons were found to be 100% identical to the *ompA* sequence of *R. africae* published in GenBank (CP001612.1).

Molted nymphs were the source of 3 strains of *R. africae*. Although dogs are rarely reported to be hosts of *A. variegatum* (*3*), a dog was the host of the tick that carried the first isolated strain. A 1,290-bp fragment of the rickettsial *gltA* gene and a 632-bp fragment of the *ompA* gene from all 3 strains were identical to the published sequence of the *R. africae* genome (CP001612.1). Multispacer typing showed that all 3 *R. africae* strains exhibited a genotype identical to that of all previously genotyped *R. africae* strains (genotype 38). To the best of our knowledge, this is the northernmost reported isolation of this pathogen in western Africa.

Taking into consideration data described in previous studies and the results of our work, we conclude that *A. variegatum* is an aggressive and abundant species of tick. The reported transovarial transmission rate of 100% for *R. africae* (*10*), the abundance of ticks, and the high percentage of ticks that are infected (*3*) increase the probability that humans will be bitten by infected ticks. *R. africae* is present in Senegal, and human infections (in tourists and indigenous populations) may be as common there as in southern Africa, but better availability of diagnostic assays is needed. Surveys of the distribution of vector ticks and rickettsiae should be performed, and target groups should be screened.
